# Critical Role of IRF-3 in the Direct Regulation of dsRNA-Induced Retinoic Acid-Inducible Gene-I (RIG-I) Expression

**DOI:** 10.1371/journal.pone.0163520

**Published:** 2016-09-23

**Authors:** Ryo Hayakari, Tomoh Matsumiya, Fei Xing, Hidemi Yoshida, Makoto Hayakari, Tadaatsu Imaizumi

**Affiliations:** 1 Department of Vascular Biology, Institute of Brain Science, Hirosaki University Graduate School of Medicine, Hirosaki, Japan; 2 Department of Pharmaceutical Science, Hirosaki University Graduate School of Medicine, Hirosaki, Japan; University of Nebraska-Lincoln, UNITED STATES

## Abstract

The cytoplasmic viral sensor retinoic acid-inducible gene-I (RIG-I), which is also known as an IFN-stimulated gene (ISG), senses viral RNA to activate antiviral signaling. It is therefore thought that RIG-I is regulated in a STAT1-dependent manner. Although RIG-I-mediated antiviral signaling is indispensable for the induction of an appropriate adaptive immune response, the mechanism underlying the regulation of RIG-I expression remains elusive. Here, we examined the direct regulation of RIG-I expression by interferon regulatory factor 3 (IRF-3), which is an essential molecule for antiviral innate immunity. We initially found that RIG-I can be induced by dsRNA in both IFN-independent and IRF-3-dependent manners. A sequence analysis revealed that the RIG-I gene has putative IRF-3-binding sites in its promoter region. Using a combination of cellular, molecular biological, and mutational approaches, we first showed that IRF-3 can directly regulate the expression of RIG-I via a single IRF-element (IRF-E) site in the proximal promoter region of the RIG-I gene in response to dsRNA. IRF-3 is considered a master regulator in antiviral signaling for the generation of type I interferons (IFNs). Thus, our findings demonstrate that RIG-I expression induced by the IRF-3-mediated pathway may serve as a crucial antiviral factor for reinforcing a surveillance system against viral invasion through the regulation of the cytoplasmic viral sensor RIG-I.

## Introduction

The innate immune system serves as the first line of defense against invading pathogens. This time-honored system is activated in the host until specific protection by the adaptive immune system is induced [1]. Upon viral infection, the nucleic acids of viruses are sensed by intracellular virus sensors, such as Toll-like receptors (TLRs) and retinoic acid-inducible gene-I (RIG-I)-like receptors (RLRs) [2]. RLRs include three members: RIG-I [3], melanoma differentiation-associated gene 5 (MDA5) [4] and laboratory of genetics physiology 2 (LGP2) [5]. RIG-I and MDA5 have a tandem caspase recruitment domain (CARD), whereas LGP2 lacks a CARD domain [6]. Following the recognition of viral RNA, RIG-I associates via a CARD-CARD interaction [7] with an adaptor protein, namely mitochondrial antiviral-signaling (MAVS) (also known as IPS-1, VISA, or Cardif), which localizes to the outer mitochondrial membrane [8]. A recent study showed that MAVS also localizes to peroxisomes, and peroxisomal MAVS triggers interferon (IFN)-regulatory factor-1 (IRF-1)-mediated antiviral signaling [9]. The interaction of RIG-I with MAVS triggers activation of the IRF-3/7 and NF-κB signaling pathways, leading to the production of type I IFNs and cytokines [10,11].

IRFs constitute a family of transcriptional factors that includes nine members (IRF-1 to IRF-9). Each member shares extensive homology in the amino-terminal DNA-binding domain, which is characterized by five tryptophan repeat elements located within the first 150 amino acids of the protein [12]. The DNA-binding domains of IRFs can bind to cis-acting DNA sequence elements, including the IRF element (IRF-E) [13] and IFN-stimulated response element (ISRE) [14]. Among IRFs, IRF-3 is a key molecule for the induction of type I IFNs in response to viral infection [15]. IRF-3 is ubiquitously expressed, and its expression is unaltered by viral infection or IFN treatment [16]. Various modifications of the IRF-3 protein are necessary for its activation. For instance, phosphorylation of the carboxyl-terminal end of IRF-3 is essential for the transduction of antiviral signaling [17]. IRF-3 can shuttle between the nucleus and the cytoplasm, but is mainly located in the cytoplasm under normal conditions [18]. After viral infection, dimerized IRF-3 can be observed in the nucleus, and this dimerized IRF-3 subsequently associates with the promoter region of IRF-E, leading to enhancements in the expression of type I IFN and cytokines.

RIG-I is a known member of IFN-stimulated genes (ISGs), and the expression of RIG-I is induced by viral infection in a type I IFN-dependent manner [2]. In this cascade, viral RNA triggers RLR-mediated IFN synthesis [19]. Therefore, the expression of RIG-I is required to accelerate RLR-mediated antiviral signaling (RLR signaling) for the induction of ISGs. In this positive feedback system for the expression of RIG-I, type I IFN-activated STAT1 initiates RIG-I induction [20]. To rapidly sense viral RNA, the induction of RIG-I should be directly regulated. However, these findings have not yet explained the mechanism through which the expression of RIG-I is regulated in response to various stimuli. The expression of the viral sensor RIG-I should be crucial for the “patrolling” of invading viruses in host cells. In fact, mice lacking RIG-I are highly susceptible to viral infection [21]. Therefore, in the present study, we explored the mechanism of early expression of RIG-I in response to non-self RNA mimicking RNA virus infection. We observed that non-self RNA rapidly induced the expression of RIG-I in a type I IFN-independent fashion in HeLa cells. We also observed a critical role of IRF-3 in non-self RNA-mediated RIG-I expression. Our findings address the direct regulation of RIG-I by IRF-3 upon viral infection.

## Materials and Methods

### Cell culture

HeLa cells were obtained from the American Type Culture Collection. U3A, U5A, and 2fTGH cells were kindly provided by G. Stark (Cleveland Clinic Foundation Research Institute). These cells were maintained in a 5% CO_2_ atmosphere at 37°C in Dulbecco’s modified Eagle’s medium (DMEM) (Sigma-Aldrich, St Louis, MO, USA) supplemented with 10% fetal bovine serum (FBS) (Sigma-Aldrich).

### Transfection

The cells were plated at a density of 0.5 × 10^5^ cells per well in a 12-well culture plate approximately 24 h prior to transfection. RNA interference (RNAi) was performed via transfection with gene-specific siRNAs or control siRNA using Lipofectamine RNAiMAX (Invitrogen, Carlsbad, CA, USA) for 48 h according to the manufacturer’s instructions. Silencer® siRNA against IRF-1 (s7501) and non-silencing control siRNA were purchased from Life Technologies. siRNA against IRF-3 (SI03117359) was purchased from Qiagen (Hilden, Germany). The cells were transfected with poly I:C using the TransFectin Lipid Reagent (Bio-Rad, Hercules, CA, USA) following the manufacturer's instructions. The cells were incubated for various durations depending on the experiment and then further analyzed.

### RNA extraction and quantitative reverse transcription-PCR (qRT-PCR)

We isolated the total RNA from the cells using an illustra RNAspin mini RNA isolation kit (GE Healthcare, Little Chalfont, United Kingdom). The total RNA (500 ng) was used as a template for single-strand cDNA synthesis using ReverTra Ace reverse transcriptase (Toyobo, Osaka, Japan) under the conditions indicated by the manufacturer. A CFX96 real-time PCR detection system (Bio-Rad) was used for the quantitative assessment of the mRNA levels of RIG-I, IRF-3, glyceraldehyde-3-phosphate dehydrogenase (GAPDH) and MAVS and the amounts of 18S rRNA. The sequences of the primers used for PCR were the following:

RIG-I-F (5’-GTGCAAAGCCTTGGCATGT-3’),

RIG-I-R (5’-TGGCTTGGGATGTGGTCTACTC-3’),

IRF3-F (5’-TACGTGAGGCATGTGCTGA-3’),

IRF3-R (5’-AGTGGGTGGCTGTTGGAAAT-3’),

GAPDH-F (5’-GCACCGTCAAGGCTGAGAAC-3’),

GAPDH-R (5’-ATGGTGGTGAAGACGCCAGT-3’),

MAVS-F (5’-ATAAGTCCDGAGGGCACCTTT-3’),

MAVS-R (5’-GTGACTACCAGCACCCCTGT-3’),

18S rRNA-F (5’-ACTCAACACGGGAAACCTCA-3’), and

18S rRNA-R (5’-AACCAGACAAATCGCTCCAC-3’).

The amplification reactions were performed with SsoAdvanced Universal SYBR Green Supermix (Bio-Rad) according to the manufacturer’s specifications. The amplification conditions were as follows: 98°C for 30 s and 40–44 cycles of 10 s at 98°C and 30 s at 58°C. After amplification, a melting curve was generated by continuously recording the fluorescence as the reaction was slowly heated from 65°C to 95°C at increments of 0.5°C over 5 s per step. The data were analyzed using CFX Manager (Bio-Rad). The mRNA levels were calculated by normalizing to the 18S rRNA amounts. The data represent the means ± SD from three independent determinations.

### Immunoblot analysis

The cultured cells were washed twice with phosphate-buffered saline (PBS, pH 7.4) and then harvested in hypotonic lysis buffer [10 mM Tris (pH 7.4), 100 mM NaCl 1.5 mM MgCl_2_, and 0.5% NP-40] containing 0.2% protease inhibitor cocktail (Sigma-Aldrich). The cell lysates were cleared by centrifugation at 12,000 x g and 4°C for 10 min. We quantified the protein concentrations in the lysate using a BCA protein assay kit (Thermo Fisher Scientific). Eight micrograms of the cell lysate were subjected to electrophoresis on an 8.0% polyacrylamide gel. To assay IRF-3 dimerization, native PAGE was performed as previously described [22]. The proteins were transferred to PVDF membranes (Millipore, Bedford, MA, USA), which were then blocked for 1 h at room temperature in TBST buffer [20 mM Tris (pH 7.4), 150 mM NaCl, and 0.1% Tween 20] containing 1% nonfat dry milk. The membranes were incubated overnight at 4°C with a primary antibody in blocking buffer (TBST-1% nonfat dry milk). The following primary antibodies were used: anti-IRF-3 antibody (Immuno-Biological Laboratories, Gunma, Japan), anti-IRF-1 antibody, anti-HSP90 antibody, anti-histone H1 antibody (Santa Cruz Biotechnology, Santa Cruz, CA, USA), and anti-β-actin antibody (Sigma-Aldrich). After five washes with TBST, the membranes were further incubated for 1 h at room temperature with a bovine anti-rabbit (Santa Cruz Biotechnology) or Zymax mouse IgG antibody conjugated to HRP (Invitrogen) at a dilution of 1:5,000 in blocking buffer. After further washes with TBST, the immunoreactive bands were visualized using the Luminata Crescendo Western HRP Substrate (Millipore). A representative result from three independent determinations is shown.

### Immunofluorescence analyses

HeLa cells grown on glass coverslips were fixed with 4% formaldehyde for 20 min, permeabilized with 0.1% Triton X-100 for 10 min, and blocked with 2% BSA for 1 h. The cells were then incubated for 1 h with anti-IRF-1 and anti-STAT1 antibodies (Santa Cruz Biotechnology). After a washing step, the cells were incubated with Alexa 488-conjugated anti-mouse IgG and Alexa 555-conjugated anti-rabbit IgG. The cells were mounted in ProLong Gold antifade reagent with DAPI (Life Technologies), and the subcellular localizations of IRF-1 and IRF-3 were visualized by confocal laser scanning microscopy (C1si, Nikon, Japan).

### Promoter activity assays

We introduced a promoter region of RIG-I that comprised nt -2,000 to +156 into pGL4.11 (Promega, Madison, WI, USA) and used genomic DNA isolated from HeLa cells. Phusion DNA polymerase (Thermo Fisher Scientific, Waltham, MA, USA) was combined with a sense primer harboring a 5’-HindIII site and an antisense primer designed with a 5’-NcoI site (both shown in lowercase font) as follows: HindIII-(-2000), 5’-AGTaagtccGCTTCCTGGGTTCAAGCGAT-3’ (sense) and NcoI-CDs, 5’-GCGGccatggCGGCCTCTGCTTGCAG-3’ (antisense). Serial deletions of the reporter constructs were further generated. The following primer pairs were used for the various constructs: “-1323”, 5’-AGTaagcttAGGAAGGGGTAATTGACAA-3’ (sense) and NcoI-CDs (antisense); “-887”, 5’-AGTaagcttGGACCCCCCATCTCACGC-3’ (sense) and NcoI-CDs (antisense); “-291”, 5’-AGTaagcttAGTaagcttAGCTAAACATAGACTTAC-3’ (sense) and NcoI-CDs (antisense); “-130”, 5’-AGTaagcttAGTAAGCTTCGCCGCTAGTTGCACTTTC-3’ (sense) and NcoI-CDs (antisense); and “-4”, 5’-AGTaagctt CCTTTAGTTATTAAAGTT-3’ (sense) and NcoI-CDs (antisense). The deletion mutant of -130 to -5 was obtained by inverse PCR using “-2000” as the template and the following primer pairs: “Δ-130 to -5”, 5’-CCTTTAGTTATTAAAGTTCCTATGCAGC-3’ (sense) and 5’-TTTATGATCTATATTTGTTTTGCTTTATAGCGC-3’ (antisense). A series of deletions of putative IRF-3-binding sites were obtained by inverted PCR to generate each single deletion using “-291” as a template. The following primer pairs were used: “ΔISRE”, 5’-GCCCGAGGCAAAACAGC-3’ and 5’-CCGCACCGAGGAAGCCC-3’; “ΔGAS”, 5’-CCCCGCCCGCCGCTAG-3’ and 5’-GCTGTTTTGCCTGGGC-3’; and “ΔIRF-E”, 5’- AGTTATTAAAGTTCCTATG-3’ and 5’-TGCAACTAGCGGCGGGCG-3’. After sequence analysis, double-deletion mutants were generated using single-deletion mutants as templates. We ultimately generated triple mutants using the double-deletion mutants as templates. Serial nucleotide deletions from the RIG-I promoter “-291” were generated using the HindIII-“-291” sense primer described above and the following antisense primers designed with a 5’-NcoI site (shown in lowercase font): 5’-GCGGccatggCCGGCACTAAAGGGAAAATCGA-3’ (+4), 5’-GCGGccatggTAAAGGGAAAATCGAAAG-3’ (+2), 5’-GCGGccatggAAGGGAAAATCGAAAGTG-3’ (-1), 5’-GCGGccatggGAAAATCGAAAGTGCAAC-3’ (-5), 5’-GCGGccatggATCGAAAGTGCAACTAGC-3’ (-9), and 5’-GCGGccatggCCGGCGCAACTAGCGGCGGGCGG-3’ (-18).

### Preparation of nuclear extracts

The cultured cells were washed twice with PBS and then harvested in hypotonic lysis buffer containing 1 mM dithiothreitol (DTT) and 100 μM phenylmethylsulfonyl fluoride (PMSF). The lysate was homogenized and cleared by centrifugation at 2,500 rpm and 4°C for 4 min. The nuclear pellets were washed twice with tNP-40-free hypotonic lysis buffer and then eluted in nuclear extract buffer [20 mM Tris-HCl (pH 8.0), 420 mM NaCl, 1.5 mM MgCl_2_, 0.2 mM EDTA, and 25% glycerol]. The protein concentrations in the extract were determined using a Quick Start Bradford protein assay kit (Bio-Rad).

### Purification of glutathione-S-transferase (GST)-IRF-3 fusion protein

Using a cDNA library from HeLa cells as a template, cDNA encoding the full-length human IRF-3 coding region was amplified by Phusion DNA polymerase with a sense primer harboring a 5’-EcoRI site and an antisense primer designed with a 5’-XhoI site as follows: 5’-CCCgaattcATGGGAACCCCAAAGCCA-3’ (sense) and 5’-CTAGActcgagTCAGCTCTCCCCAGGGCC-3’ (antisense). The amplicon was then inserted into the pGEX5x-1 (GE healthcare, Little Chalfont, United Kingdom) vector to generate a GST fusion protein. Using this vector as the template, IRF-3(5D), a constitutively active mutant of IRF-3, was generated as previously reported [23]. The following primer pairs were used to generate the mutations in the GST-IRF3 wild-type (WT) sequence: “1st round PCR", 5’- GACAACGACCACCCACTCTCCCTCACCT -3’ (sense) and 5’- AATGTGCAGGTCCACAGTA -3’ (antisense); “2nd round PCR", 5’- GACCTCGACGACGACCAGTACAAGGCCTA (sense) -3’ and 5’- GAGTGGGTGGTCGTTGTCA -3’ (antisense). Protein expression was induced by the addition of 0.3 mM isopropyl β-D-1-thiogalactopyranoside (IPTG). After incubation at room temperature for 3 h, the cells were lysed with FLAG IP lysis buffer [50 mM Tris-HCl (pH 7.4), 150 mM NaCl, 1 mM EDTA, and 1% Triton X-100] and purified using glutathione-Sepharose 4B (GE Healthcare) and Amicon Ultra Centrifugal Filter devices (30K) (Millipore).

### Electrophoretic mobility shift assay (EMSA)

An EMSA was performed using nuclear protein extracts obtained as described above. Synthetic double-stranded oligonucleotides containing WT RIG-I-IRF-E (sense oligonucleotide, 5’-GTTGCACTTTCGATTTTCCCTTTAGTTATTAAAG-3’, and antisense oligonucleotide, 5’-GGAACTTTAATAACTAAAGGGAAAATCGAAAGTG-3’) or mutant RIG-I-IRF-E (sense, 5’-GTTGCACTTTCGATgggaCCTTTAGTTATTAAAG-3’, and antisense, 5’-GGAACTTTAATAACTAAAGGtcccATCGAAAGTG-3’) were labeled with digoxigenin (DIG) using a DIG gel shift kit (Roche, Basel, Switzerland). Five micrograms of proteins in the nuclear lysate or GST-fusion proteins were incubated with 8 pmol of the labeled DNA probe. The binding reactions were subjected to electrophoresis on a 6.0% DNA retardation gel (Invitrogen). The probes were then transferred to a positively charged Nylon membrane (Roche), and the membrane was then crosslinked by exposure to UV (312 nm) for 5 min. Detection of the labeled probes was accomplished using a DIG nucleic acid detection kit (Roche). A representative result from three independent determinations is shown. For supershift experiments, the nuclear extracts were pre-incubated with rabbit anti-IRF-3 (Active Motif, Carlsbad, CA, USA) or normal rabbit antibodies prior to addition of the labeled probes.

### Chromatin immunoprecipitation (ChIP)

HeLa cells were fixed with 1.0% formaldehyde for 10 min at room temperature (RT) and neutralized with 1.5 M glycine for 5 min at RT. The fixed cells were washed twice with ice-cold PBS and then harvested in SDS lysis buffer [50 mM Tris (pH 8.0), 10 mM EDTA (pH 8.0), 1% SDS] containing 0.2% protease inhibitor cocktail (Sigma-Aldrich). The cell lysates were sonicated at 4°C and cleared by centrifugation at 15,000 rpm and 10°C for 10 min. Immunoprecipitation was performed using Dynabeads protein G (Veritas, Tokyo, Japan) with rabbit anti-IRF-3 (Active Motif) or control rabbit IgG (Santa Cruz). The proximal RIG-I promoter region from -80 to +2 was amplified by qPCR using the following primer pair: 5’- GGAGGGAAACGAAACTAGCC -3’ (sense) and 5’- CGGAGCTGCATAGGAACTTT -3’ (antisense).

### Cell viability assay

Cell viability assays were performed using the CellTiter-Glo luminescent cell viability assay kit (Promega) according to the manufacturer’s specifications. The relative luminescent units were normalized to the total protein amounts in each sample.

### Statistics

Statistical analyses were performed using Student’s t-test. Differences with †P<0.05 or *P<0.01 were considered significant.

## Results

### Type I IFN-independent RIG-I expression in response to dsRNA

**Fig 1:**
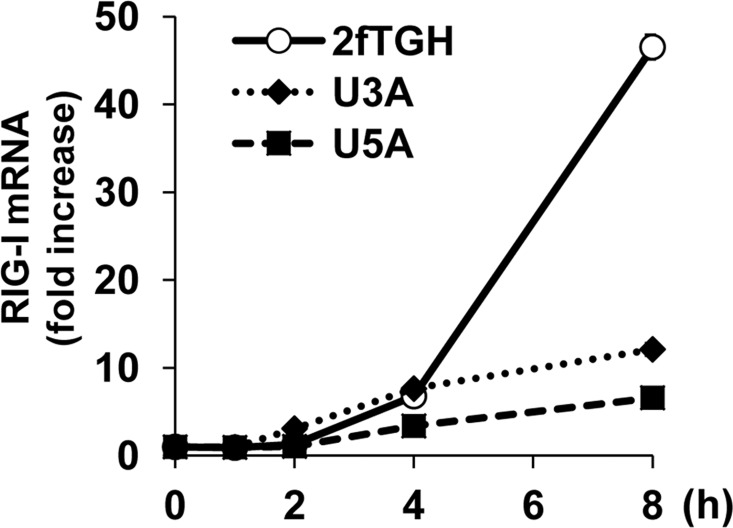
STAT1- and type I IFN-independent RIG-I expression in response to dsRNA. 2fTGH, U3A, and U5A cells were transfected with poly I:C (100 ng) and incubated for up to 8 h. The expression levels of RIG-I were determined by quantitative RT-PCR. The means (±SD) of three experiments are shown.

Although RIG-I is an ISG, the reason why RIG-I is induced in response to RLR signaling remains to be elucidated. Therefore, we first examined the molecular mechanism underlying the induction of RIG-I expression through the activation of RLR signaling. Polyinosinic:polycytidylic acid (poly I:C) is a synthetic double-stranded RNA (dsRNA) recognized by various pattern recognition receptors (PRRs), including TLR3, RIG-I, and MDA-5, and this recognition subsequently activates antiviral signaling [3]. We previously showed that the transfection of cells with poly I:C selectively activates RLR signaling but not TLR signaling [24]. The transfection of HeLa cells with poly I:C upregulates RIG-I mRNA in a time-dependent manner (S1A Fig). STAT1 has been demonstrated to serve as a crucial transcriptional factor for the induction of RIG-I [25]. Indeed, both type I and type II IFNs induce the expression of RIG-I in HeLa cells (S1B Fig). We then asked whether the induction of RIG-I expression in response to dsRNA is absolutely dependent on STAT1. To address this question, we used STAT1-null U3A cells, type I IFN receptor (IFNAR)-null U5A cells, and their parental 2fTGH cells [26] to examine the effects of STAT1 and type I IFN on the expression of RIG-I in response to RLR signaling. The transfection of poly I:C stimulated RIG-I expression in both STAT1- and IFNAR-presenting 2fTGH cells (Fig 1). We initially found that RLR signaling can induce the expression of RIG-I even in the absence of STAT1 or IFNAR and that the kinetics of RIG-I expression was similar under all conditions for up to 4 hours (Fig 1, U3A and U5A cells). By contrast, eight hours after poly I:C transfection, 2fTGH cells expressed approximately 4- and 7-fold higher levels of RIG-I mRNA than U3A and U5A cells, respectively. These increases in RIG-I expression in 2fTGH cells are thought to be due to the positive feedback of type I IFN induced by RLR signaling. A relatively rapid (< 4 h) induction of RIG-I was observed in U3A and U5A cells suggesting the type I IFN-independent RIG-I expression.

### Influence of IRF-1 and IRF-3 on RLR signaling-mediated RIG-I expression

We therefore hypothesized that RLR signaling may directly regulate the expression of RIG-I. A previous study showed that IRF-1 regulates the expression of RIG-I in response to poly I:C [27].

**Fig 2:**
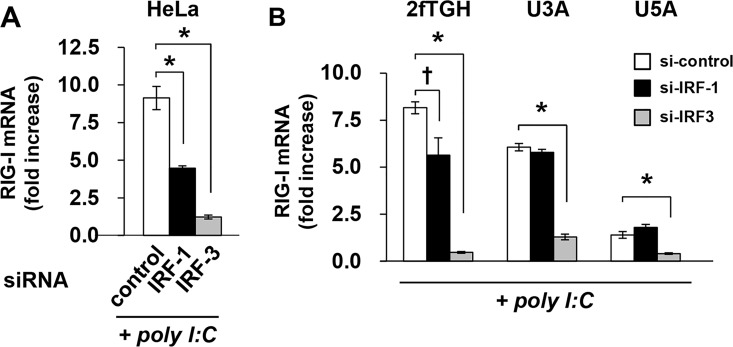
STAT1- and type I IFN-independent RIG-I expression requires IRF3. HeLa (A), and 2fTGH, U3A, and U5A cells (B) were transfected with siRNA against IRF-1 or IRF-3 or control siRNA for 48 h and then transfected with poly I:C (100 ng) for 4 h. The expression levels of RIG-I were determined by quantitative RT-PCR. The means (±SD) of three experiments are shown; †*P* < 0.05, *P < 0.01.

In our system, we also confirmed that IRF-1 silencing inhibited the RLR signaling-induced expression of RIG-I in HeLa and 2fTGH cells (Fig 2A and 2B). However, this effect was not observed in U3A and U5A cells (Fig 2B), indicating that the activation of IRF-1 in RLR signaling is dependent on STAT1 and IFNAR signaling.

**Fig 3:**
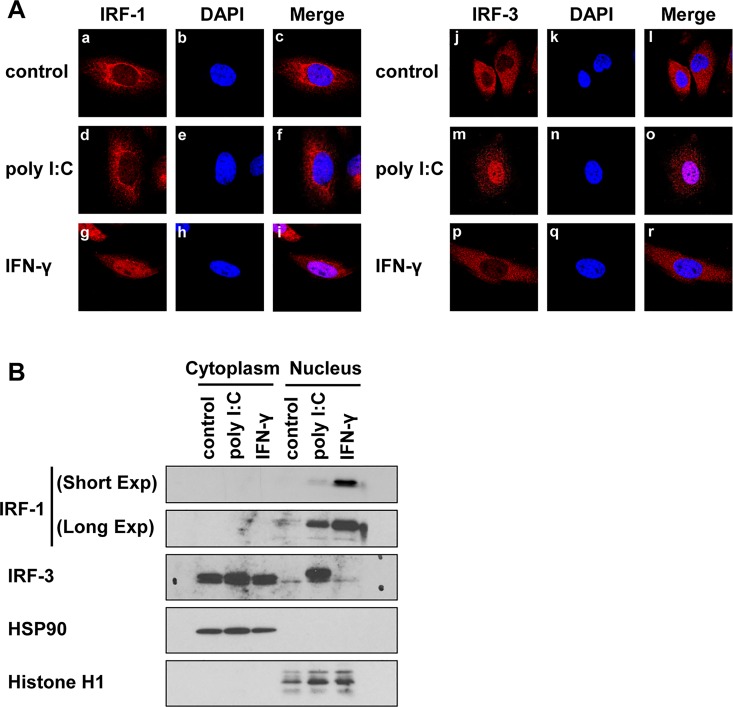
Intracellular localization of IRF-1 and IRF-3. (A) HeLa cells were subjected to the following treatments: control (a-c, j-l), transfected with poly I:C (100 ng, for 4 h) (d-f, m-o), or treated with IFN-γ (2 ng/mL, for 4 h) (g-i, p-r). The cells were then fixed with 4% paraformaldehyde and co-stained for IRF-1 (red, left panels) or IRF-3 (red, right panels). Nuclei were counterstained with DAPI (blue). A representative result for five random fields is shown. (B) HeLa cells were transfected with poly I:C (100 ng, for 4 h) or treated with IFN-γ (2 ng/mL, for 4 h). The cells were then harvested in lysis buffer and fractionated. The cell extracts were subsequently subjected to SDS-PAGE, blotted, and probed with anti-IRF-1, anti-IRF-3, anti-HSP90, or anti-histone H1 antibodies. The results are representative of three independent experiments.

Activation of RLR signaling induces the expression of IRF-1; however, a low nuclear accumulation of IRF-1, even in cells expressing nuclear NF-κB, was observed, whereas the IRF-1 activator IFN-γ was found to induce a rapid and marked nuclear accumulation of IRF-1 (Fig 3A). A biochemical analysis additionally revealed low levels of IRF-1 expression in untreated cells and that IFN-γ results in the marked accumulation of IRF-1 in the nucleus (Fig 3B). In contrast to the effects of IFN-γ stimulation, a limited nuclear localization of IRF-1 was observed after poly I:C transfection. These findings indicate that the effects of RLR signaling on the activation of IRF-1 appear to be limited. IRF-3 is a cardinal molecule in RLR signaling for the induction of type I IFN, and this induction subsequently stimulates ISGs [2]. IRF-3 is predominantly located in the cytoplasm and translocates to the nucleus in response to activation of RLR signaling (Fig 3). We then investigated the direct role of IRF-3 in the induction of RIG-I expression. To this end, we confirmed the basal expression of IRF-3 and the efficiency of the knockdown of IRF-3 in U3A, U5A, and 2fTGH cells (S2A Fig). The silencing of IRF-3 markedly decreased the expression level of RIG-I in HeLa and 2fTGH cells after poly I:C transfection (Fig 2). In addition, IRF-3 knockdown significantly suppressed RLR signaling-mediated RIG-I expression in U3A and in U5A cells (Fig 2B). We confirmed that knockdown of IRF-3 did not affect cell viability (S2B Fig), thus indicating that the inhibition of RIG-I expression was not due to cell damage by IRF-3 silencing. Collectively, these results show that IRF-3 directly regulates the expression of RIG-I in response to RLR signaling. We noted that the transfection of poly I:C induced the dimerization of IRF-3 in 2fTGH, U3A, and U5A cells, indicating that RLR signaling is intact in these cells (S3 Fig).

### Role of the IRF element in the dsRNA-induced expression of RIG-I

**Fig 4:**
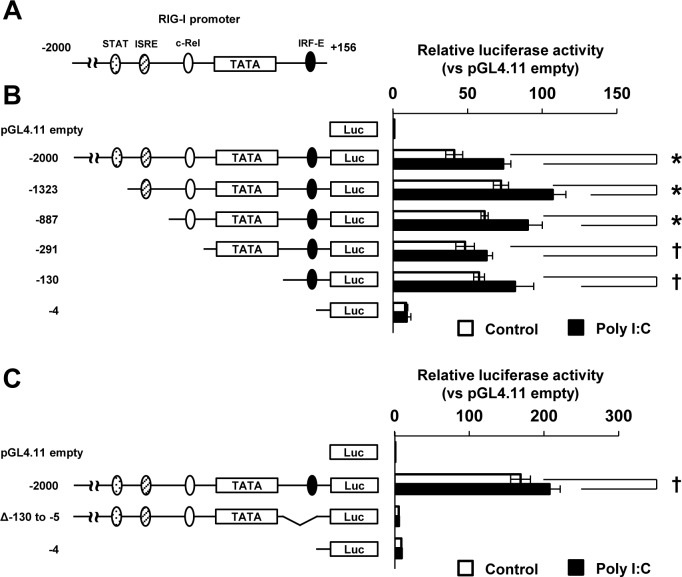
Promoter analysis of the human RIG-I gene. (A) Putative consensus sequences of STAT1, ISRE, c-Rel and IRF-E on the RIG-I promoter are shown. (B) and (C) HeLa cells were co-transfected with pGL4.11 (empty) or serial human RIG-I luciferase reporter constructs and Renilla luciferase expression vector (pGL4.74) for 24 h. The cells were further transfected with poly I:C (100 ng) for 4 h. The reporter activities are shown as relative values, specifically ratios of the firefly luciferase activities driven by the RIG-I promoters to the Renilla luciferase activities. The means (±SD) of three experiments are shown; †*P* < 0.05, *P < 0.01.

To elucidate the transcriptional regulation of constitutively expressed RIG-I, we identified consensus elements for transcription factors in the RIG-I promoter region. We cloned the 2-kb RIG-I promoter located upstream from the transcriptional start site. Through computational analysis, putative binding sites for STAT1 (-1331/-1323), ISRE (-901/-887), c-Rel (-306/-297), and IRF-E (-17/-5) were identified in the RIG-I promoter region (Fig 4A). To characterize the role of these transcriptional factors, we constructed a luciferase reporter vector containing the 2-kb RIG-I promoter. We then generated luciferase reporter vectors with serially truncated forms of the RIG-I promoter region as illustrated in Fig 4B. The luciferase activity in HeLa cells transfected with the vector containing the 2-kb RIG-I promoter sequence was approximately 45-fold higher compared with that observed in the cells transfected with an empty vector (Fig 4B). Deletions of the STAT1-binding site (-1323), ISRE (-887), or c-Rel-binding site (-291) from the 2-kb RIG-I promoter exerted an insignificant effect on the luciferase activities of the transfected cells (Fig 4B). In contrast, deletion of the IRF-E markedly reduced the promoter activity. In addition, the deletion of -130/+5 from the 2-kb RIG-I promoter eliminated luciferase activity in response to poly I:C (Fig 4C).

**Fig 5:**
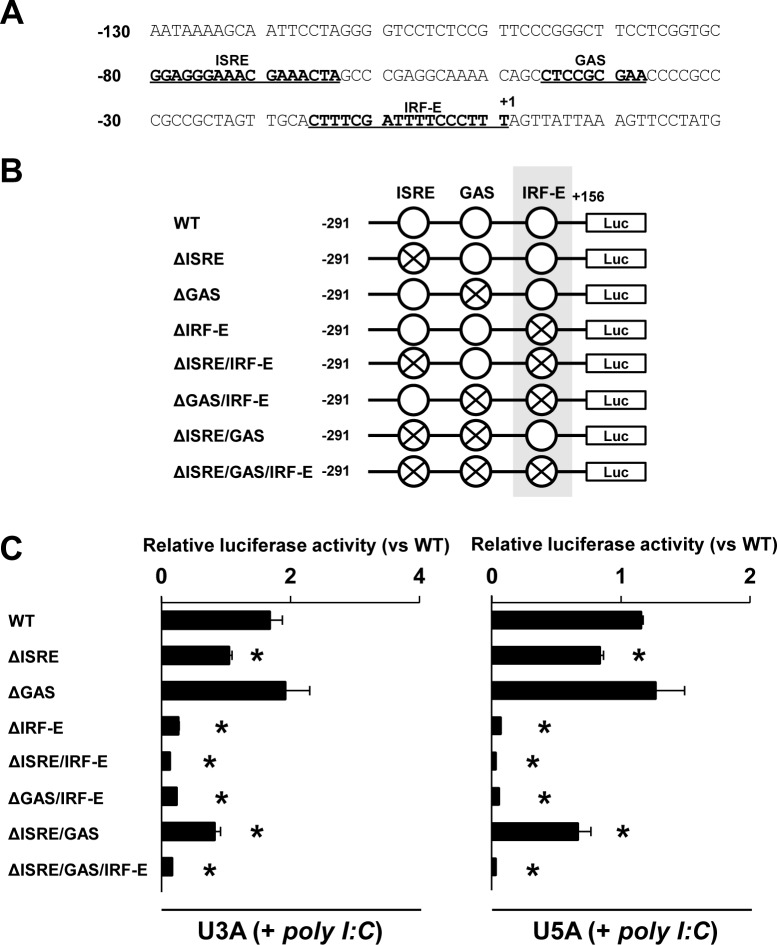
IRF-E on the RIG-I promoter regulates the transcriptional activity of RIG-I. (A) Putative consensus sequences of ISRE, GAS and IRF-E within the proximal region of the RIG-I promoter are shown. (B) A series of single (double or triple) deletion constructs on the RIG-I promoter is shown. (C) U3A and U5A cells were co-transfected with the RIG-I deletion constructs and a renilla luciferase expression vector as shown in (B); †*P* < 0.05, *P < 0.01.

Because the -130/-5 proximal sequence affected the promoter activity, we further searched the binding elements in detail and found putative ISRE (-78/-64) and GAS (-46/-38) consensus sequences in proximity to the region upstream of IRF-E (-16/+1) on the RIG-I promoter (Fig 5A). We searched murine RIG-I promoter sequences in the NCBI database and compared the human ISRE-GAS-IRF-E region (-233/+161) and murine equivalent region (-200/+132). As shown in S4 Fig, the ISRE and IRF-E sites were conserved in the murine RIG-I promoter region, whereas GAS was not observed. To evaluate the influence of each element on the promoter activity, we generated a series of deletion constructs in the proximal region of the RIG-I promoter, as illustrated in Fig 5B. We then transfected those constructs into STAT1-null U3A or IFNAR-null U5A cells to exclude the effect of type I IFN that is induced by dsRNA. In both U3A and U5A cells, the deletion of ISRE decreased the poly I:C-induced promoter activity to approximately 20%, whereas the deletion of GAS did not affect the luciferase actively. In contrast, the deletion of IRF-E almost completely suppressed the poly I:C-induced promoter activity (Fig 5C). Although the deletion of both ISRE and GAS did not result in complete elimination of luciferase activity, the deletion of these two elements in combination with IRE-E did not rescue the luciferase activity. Taken together, these results define IRF-E as the cardinal transcriptional factor-binding element for RIG-I expression.

### Association of IRF-3 with IRF-binding site in the RIG-I promoter

**Fig 6:**
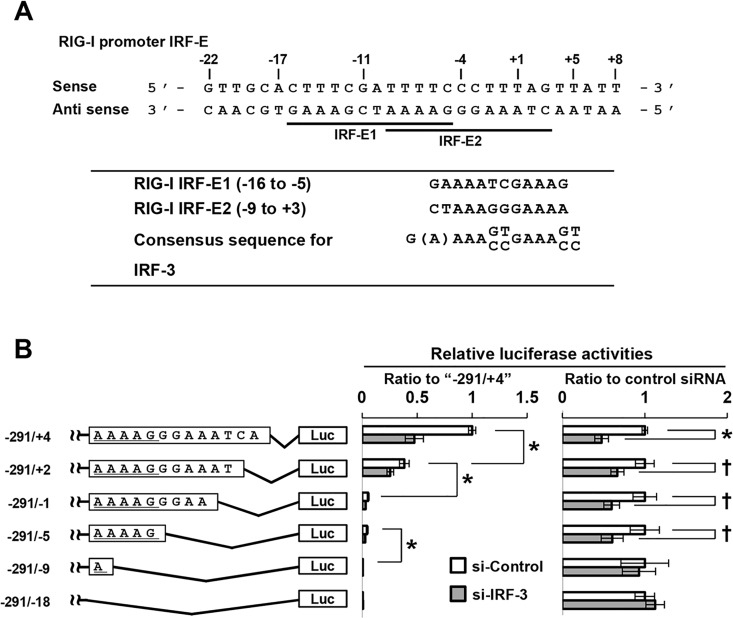
Critical role of IRF-E on the IRF3-mediated activation of the RIG-I promoter. (A) The nucleotide sequence of the RIG-I promoter (-22 to +8) is shown, and putative IRF-3-binding sites are underlined. The numbers show the positions relative to the transcription start site (+1). (B) The luciferase activities driven by vectors with serial truncations of the IRF-E in the RIG-I promoters (left panel) were analyzed (center panel). The right panel shows the effect of IRF-3 knockdown on the promoter activities of RIG-I promoters with different lengths; †*P*< 0.05, *P < 0.01.

We then investigated the functional association between the RIG-I-IRF-E site and IRF-3. To this end, we further constructed a series of truncated forms of reporter vectors based on the IRF-E. The consensus sequence for IRF-E is G(A)AAAXXGAAAXX [13]. As shown in Fig 6A, two putative IRF-E sites were found in the promoter region of RIG-I, and part of the sequences (AAAAG) overlapped. The serial truncations of IRF-E2 from proximal nucleotides gradually decreased the luciferase activity in response to poly I:C, and removal of the overlapping site completely abolished the luciferase activity (Fig 6B, open bar). These findings indicate that IRF-E2, rather than IRF-E1, is essential for the constitutive expression of RIG-I. We further examined the effect of IRF-3 on IRF-E1 in the RIG-I promoter. The silencing of IRF-3 suppressed the IRF-E-mediated luciferase reporter activity, and a similar effect was observed after deletion of the seven nucleotides (GGAAATC) proximal to IRF-E2 (Fig 6B, right panel). This effect eventually disappeared after removal of the overlapping elements from the reporter construct. These results suggest that the overlapping site is a critical element for the IRF-3-mediated constitutive expression of RIG-I.

**Fig 7:**
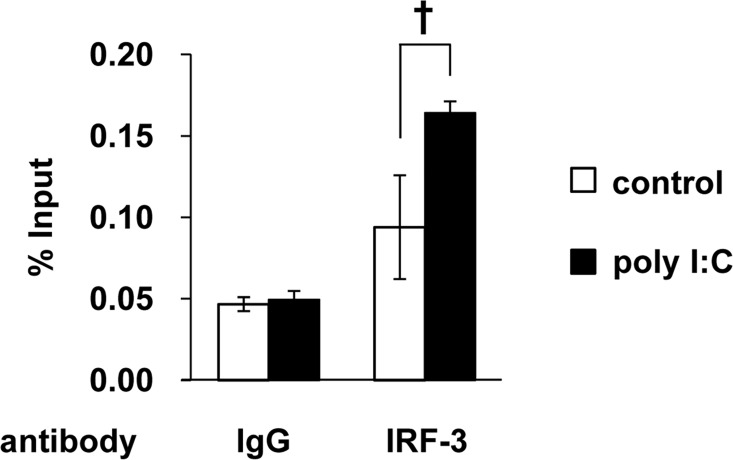
IRF-3 binding to the RIG-I promoter *in vivo*. HeLa cells were transfected with poly I:C (100 ng) for 4 h and fixed; the DNA was then fragmented. Chromatin immunoprecipitation was performed using an anti-IRF-3 antibody or control IgG. The means (±SD) of three experiments are shown. †*P* < 0.05

IRF-3 is predominantly located in the cytoplasm and is phosphorylated and translocated to the nucleus from the cytoplasm upon viral infection [17]. Indeed, a large amount of IRF-3 was found in the nuclear fraction under poly I:C-transfected conditions (Fig 3B). Therefore, we asked whether nuclear IRF-3 can induce the expression of RIG-I. To examine the association of the IRF-3 protein with the IRF-E of the RIG-I promoter, we performed a ChIP assay using an anti-IRF-3 antibody and PCR primers designated to amplify the IRF-E site of the RIG-I promoter region. The ChIP assay demonstrated that IRF-3 associated with IRF-E in the RIG-I promoter in response to the addition of poly I:C (**Fig 7**).

**Fig 8:**
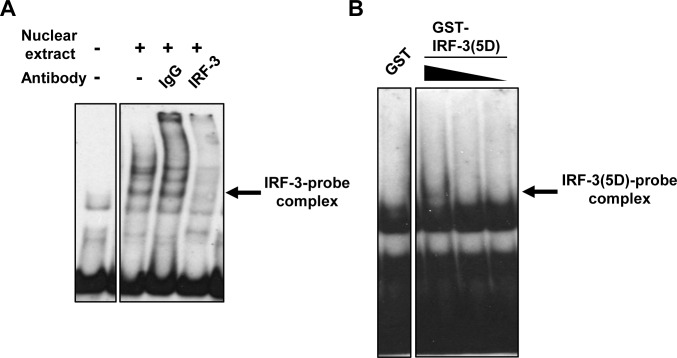
IRF-3 binds to IRF-E in the RIG-I promoter. (A) EMSA was performed using a DIG-labelled RIG-I IRF-E wild-type (WT) probe. Nuclear extracts were prepared from poly I:C-transfected HeLa cells. For the supershift assay, rabbit anti-IRF-3 antibody was pre-incubated with the reaction mixture. (B) The labeled RIG-I IRF-E-WT probe was combined with 500 nM (r)GST or (r)GST-IRF-3(5D) fusion protein at various concentrations (500 nM, 125 nM, and 50 nM; wedges). Arrows indicate the IRF-3-oligonucleotide probe complex.

To examine the detailed mechanism by which activated IRF-3 interacts with RIG-I-IRF-E, we performed an EMSA using DIG-labeled RIG-I-IRF-E as the oligonucleotide probe. The EMSA was performed using nuclear extracts prepared from poly I:C-transfected HeLa cells. Addition of the nuclear extract shifted the bands, showing an oligonucleotide-protein complex (Fig 8A). The complex disappeared after addition of an anti-IRF-3 antibody to the EMSA reaction, whereas control IgG did not influence the complex, suggesting that IRF-3 protein forms a complex with the oligonucleotide. To confirm whether IRF-3 directly binds to the IRF-E of the RIG-I promoter, we generated recombinant GST-fusion of a constitutively active form of IRF-3 [GST-IRF-3(5D)] [28] and applied the fusion protein to the oligonucleotide. Control GST protein failed to shift with the IRF-E oligonucleotides. In contrast, GST-IRF-3(5D) formed a complex with the oligonucleotide in a GST-IRF-3concentration-dependent manner (Fig 8B). Thus, our data show that IRF-3 directly associates with the IRF-E of the RIG-I promoter.

**Fig 9:**
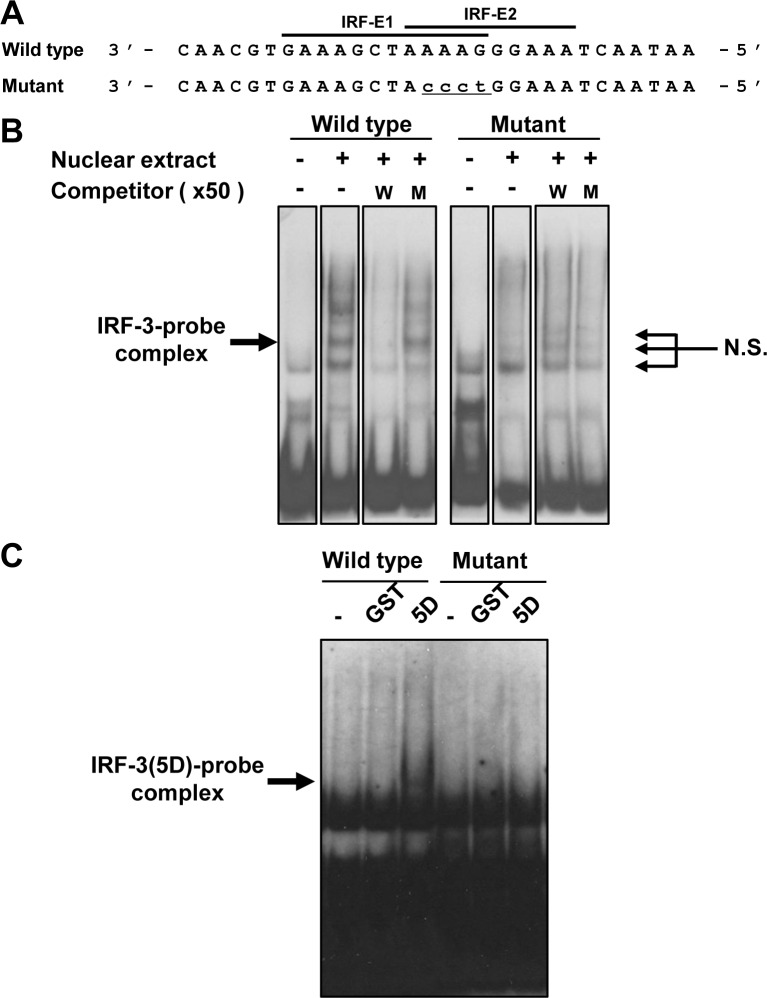
Characterization of RIG-I-IRF-E for IRF-3 binding. (A) Description of oligonucleotide probes of wild-type (WT) (-22 to +8) and mutant (MT) RIG-I IRF-E are shown. Putative IRF-3-binding sites are indicated in lowercase and underlined. (B) The RIG-I IRF-E-WT and RIG-I IRF-E-MT probes were mixed with the nuclear extract as described in Fig 6, and an EMSA was then performed. A competition assay was performed based on the addition of a 50-fold molar excess of unlabeled WT or MT probes. (C) The labeled WT or MT probes were combined with recombinant (r)GST or (r)GST-IRF-3(5D) fusion protein at various concentrations (500 nM). Arrows indicate the IRF-3-oligonucleotide probe complex. N.S.: non-specific signal.

Because our reporter activity analysis showed that the overlapping site of IRF-E is essential for the IRF-3-mediated induction of the constitutive expression of RIG-I, we examined the role of the overlapping site in regulating IRF-3-IRF-E binding. The mutated overlapping element in IRF-E (Fig 9A) was analyzed through an EMSA with the poly I:C-transfected nuclear extract (Fig 9B). The IRF-3-probe complex disappeared after addition of wild-type (WT) cold competitor, whereas competition with mutant (MT) unlabeled probe failed to eliminate the IRF-3-WT oligonucleotide complex. In addition, the IRF-E-MT oligonucleotide probe failed to form a complex with IRF-3. A study using GST-IRF-3 further showed that the mutant oligonucleotide probe failed to bind to GST-IRF-3 (Fig 9C). Collectively, these data demonstrate the ability of IRF-3 to bind to RIG-I-IRF-E and the requirement of the overlapping site of IRF-E for binding with IRF-3.

## Discussion

In the present study, we observed direct regulation of RIG-I expression by IRF-3, which is activated in response to non-self RNA. PRRs are diverse receptors that recognize pathogenic molecular patterns to activate anti-pathogenic innate immunity. Of the diverse function of PRRs, TLRs and RLRs serve as sensors for viral nucleic acids [2]. Because the immune reaction serves as the first line of host defense, dysregulation of PRR-mediated innate immune signaling results in a variety of diseases, particularly infectious diseases. Indeed, loss of functions or deficiencies of PRRs have been shown to be involved in immune deficiency. For instance, TLR3 deficiency in patients is associated with herpes simplex encephalitis [29]. Thus, PRRs likely need to be appropriately expressed in host cells in the presence of a microbe. TLR3 serves as a sensor for dsRNA and RIG-I. Kato et al. clearly showed that dsRNA activates RLR signaling in conventional dendritic cells (DCs) and TLR signaling in plasmacytoid DCs [30], indicating that the cell type-specific expression of PRRs is required to activate the antiviral innate immune response. Within the human leukocyte lineages, the expression of TLR3 is restricted to DCs [31], whereas in non-professional cells, RLRs serve as viral sensors. IFN-β is known to enhance the transcriptional activities of TLR3 [31] and TLR9 [32]. IFN-β, which is also known as a fibroblast IFN, is rapidly released from a virus-infected cell via the activation of RLR signaling to exert antiviral protection to neighboring cells. The induction of TLRs requires the activation of IRF-1 and STAT1 [31,32]. Consistent with the results of a previous study [25], our findings show that STAT1 is required for the enhanced expression of RIG-I in response to RLR signaling. In contrast, the role of IRF-1 in the regulation of RIG-I is limited; specifically, the involvement of IRF-1 in RLR signaling-mediated RIG-I expression is likely to depend on STAT1 (Fig 2B), whereas IRF-3 affects the expression of RIG-I in a STAT1-independent manner. The findings described in this manuscript indicate a novel role for IRF-3 in the RLR signaling-induced expression of RIG-I.

Mice and humans exhibit significant differences in immune system development, activation, and response to challenge, and these differences are found in both the innate and the adaptive arms of the immune system [33] and are partially due to differences in transcriptional regulation between mice and humans. For instance, the human TLR3 gene has a TATA-like element, whereas murine TLR3 promoters do not have any TATA elements [31]. A detailed analysis of the murine RIG-I promoter has not yet been reported. Our present data reveal a regulatory region proximal to the human RIG-I gene that is partially similar to that of the human TLR3 gene. Similar to its location in the human TLR3 gene, IRF-E is located downstream of the TATA-like box in the RIG-I gene (Fig 4A). GAS is also observed in the proximal promoter regions of the RIG-I and TLR3 genes. In contrast, ISRE is located in the proximal region of RIG-I but not in that of TLR3. Our sequence analysis indicates that the RIG-I promoter region is more responsive to IFNs than TLR3. In fact, the level of RIG-I is more extensively upregulated by IFNs than the level of TLR3 [31,34]. IRF-3 has an N-terminal DNA-binding domain (NTD) and a C-terminal transactivation domain (CTD), which are connected by a proline-rich linker containing a nuclear export signal sequence [17]. Upon viral infection, IRF-3 is activated by the phosphorylation of serine/threonine, leading to its translocation to the nucleus and dimerization [12]. In contrast, latent IRF-3 uses an autoinhibitory mechanism to suppress its transactivation [35]. A study using phosphomimetic mutants of IRF-3 showed higher affinity to single IRF-E compared with WT IRF-3, whereas the phosphorylated mutants presented decreased affinity for DNA [36]. The multiple phosphomimetic mutants that resulted in IRF-3 dimerization further revealed strong binding to DNA with two IRF-Es. These findings clearly show the ability of activated IRF-3 to bind the overlapping IRF-E site in the proximal region of the RIG-I promoter (Fig 7B). Thus, our data suggest the critical role of IRF-3 in the regulation of RIG-I expression.

**Fig 10:**
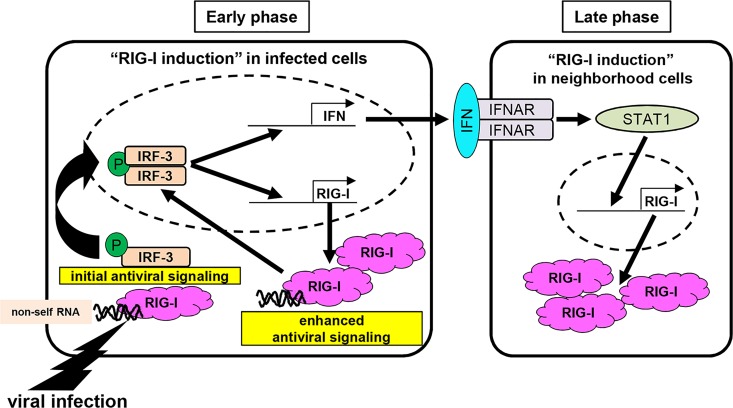
Proposal model for the direct role of IRF-3 in both constitutive and induced RIG-I expression. Upon viral infection, a low level of constitutively expressed RIG-I recognizes viral RNA, inducing the cells to reach an antiviral state. In the antiviral state, IRF-3 is phosphorylated in response to RLR signaling and translocates to the nucleus to induce type I IFNs. Our findings indicate that activated IRF-3 is also able to directly enhance the expression of RIG-I to enhance antiviral signaling. Infected cells produce IFNs, which subsequently activate STAT1, leading to the robust expression of RIG-I in neighboring cells.

In conclusion, the expression of RIG-I is regulated by IRF-3 through an IFN-dependent and IFN-independent pathway. In the early phase after viral infection, the rapid induction of RIG-I, as represented by an increased amount of cytoplasmic viral sensor, would contribute to the enhancement of antiviral signaling (Fig 10). Compared with the autocrine loop regulated by the type I IFN system, the direct induction of RIG-I by IRF-3 is likely suitable for the rapid induction of RIG-I through the RLR-IRF3 axis observed in infected cells. Our findings underscore that IRF-3 is crucial for inducing RLR signaling-mediated antiviral immunity by regulating the expression level of RIG-I.

## Supporting Information

S1 Fig Time-course expression of RIG-I. HeLa cells were transfected with poly I:C (100 ng) for up to 4 h (A) or treated with □ r(h)IFN-α2b (200 pg/mL) or ■ r(h)IFN-γ (2 ng/mL) for up to 8 h (B). The expression levels of RIG-I were determined by quantitative RT-PCR. The means (±SD) of three experiments are shown.
(TIF)

S2 Fig Knockdown efficiency of IRF-3. (A) 2fTGH, U3A, and U5A cells were transfected with IRF-3 siRNA or control siRNA. The cell extracts were subsequently subjected to SDS-PAGE and immunoblotted with anti-IRF-3 or anti-actin antibody. The results are representative of three independent experiments. (B) HeLa cells were transfected with IRF-3 siRNA or control siRNA. Forty-eight hours after transfection, cell viabilities were measured as described in the *Materials and Methods*. The means (±SD) of three experiments are shown. N.S.: not significant.
(TIF)

S3 Fig Dimerization of IRF-3 in response to RLR signaling. 2fTGH, U3A and U5A cells were transfected with poly I:C (100 ng) for up to 8 h and then harvested, and the lysates were subjected to native-PAGE or SDS-PAGE. Immunodetection was performed using an anti-IRF-3 antibody.
(TIF)

S4 Fig Comparison of the proximal promoters of human and murine RIG-I. The proximal RIG-I promoters of human (-223 to +161) and mouse (-200 to +132) are shown. The putative consensus sequences of ISRE, GAS, IRF-E are highlighted. The asterisks indicate the transcription start site.
(TIF)
