# Supplementary figures and images for: Critical Role of IRF-3 in the Direct Regulation of dsRNA-Induced Retinoic Acid-Inducible Gene-I (RIG-I) Expression
Source: PLoS One. 2016 Sep 23;11(9):e0163520. doi: 10.1371/journal.pone.0163520 (PMC5035021; doi:10.1371/journal.pone.0163520)

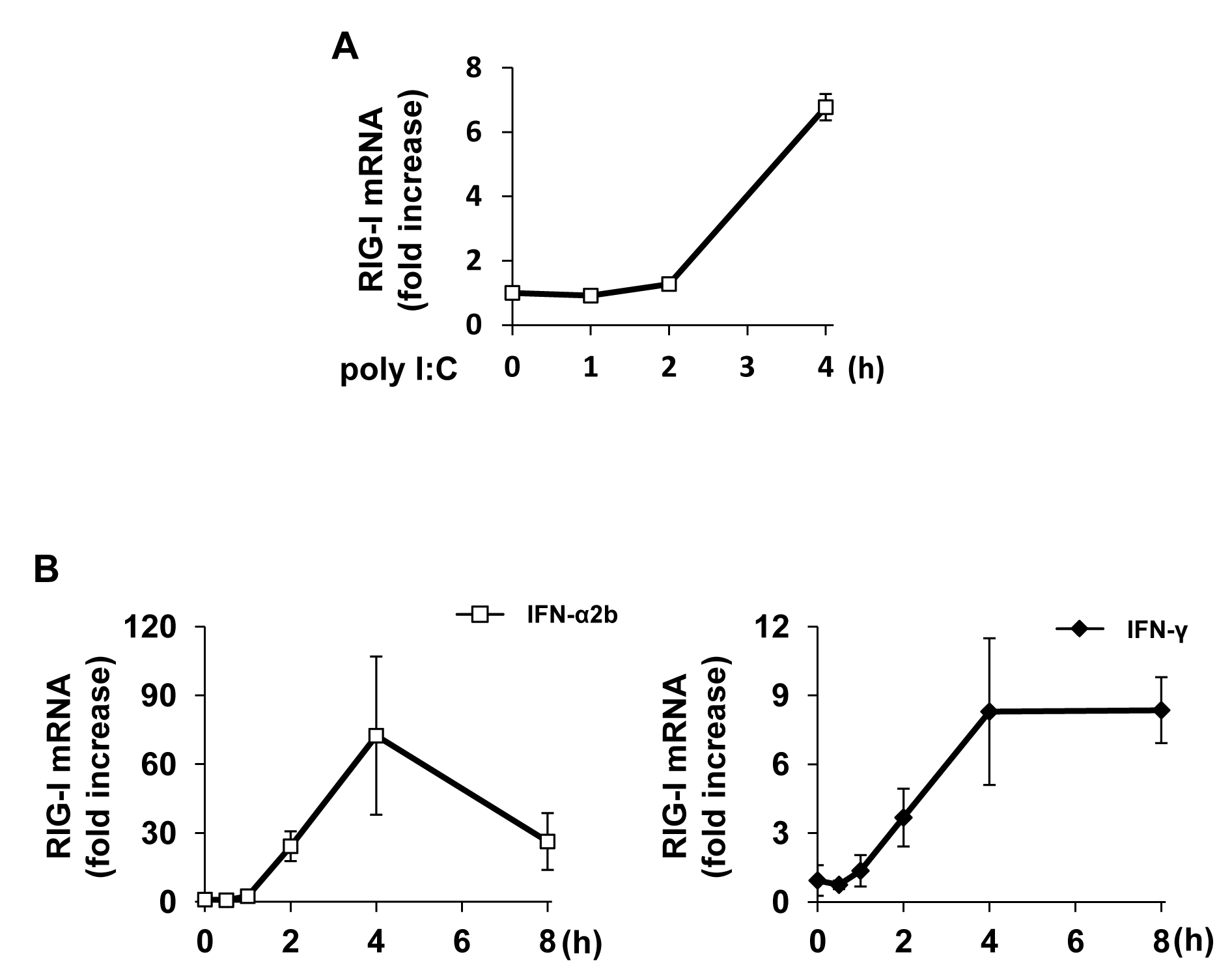

Supplement: S1 Fig — HeLa cells were transfected with poly I:C (100 ng) for up to 4 h (A) or treated with □ r(h)IFN-α2b (200 pg/mL) or ■ r(h)IFN-γ (2 ng/mL) for up to 8 h (B). The expression levels of RIG-I were determined by quantitative RT-PCR. The means (±SD) of three experiments are shown. (TIF) [file pone.0163520.s001.tif]

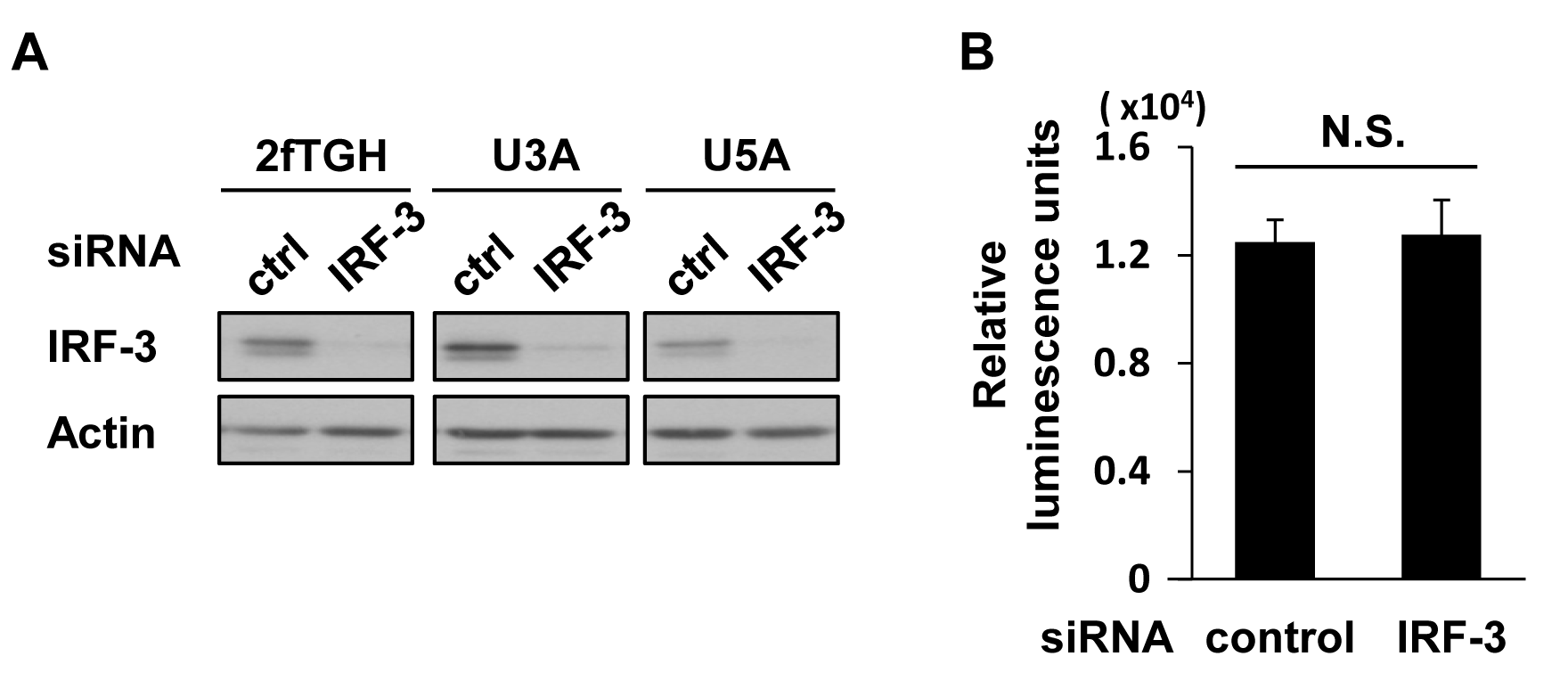

Supplement: S2 Fig — (A) 2fTGH, U3A, and U5A cells were transfected with IRF-3 siRNA or control siRNA. The cell extracts were subsequently subjected to SDS-PAGE and immunoblotted with anti-IRF-3 or anti-actin antibody. The results are representative of three independent experiments. (B) HeLa cells were transfected with IRF-3 siRNA or control siRNA. Forty-eight hours after transfection, cell viabilities were measured as described in the Materials and Methods. The means (±SD) of three experiments are shown. N.S.: not significant. (TIF) [file pone.0163520.s002.tif]

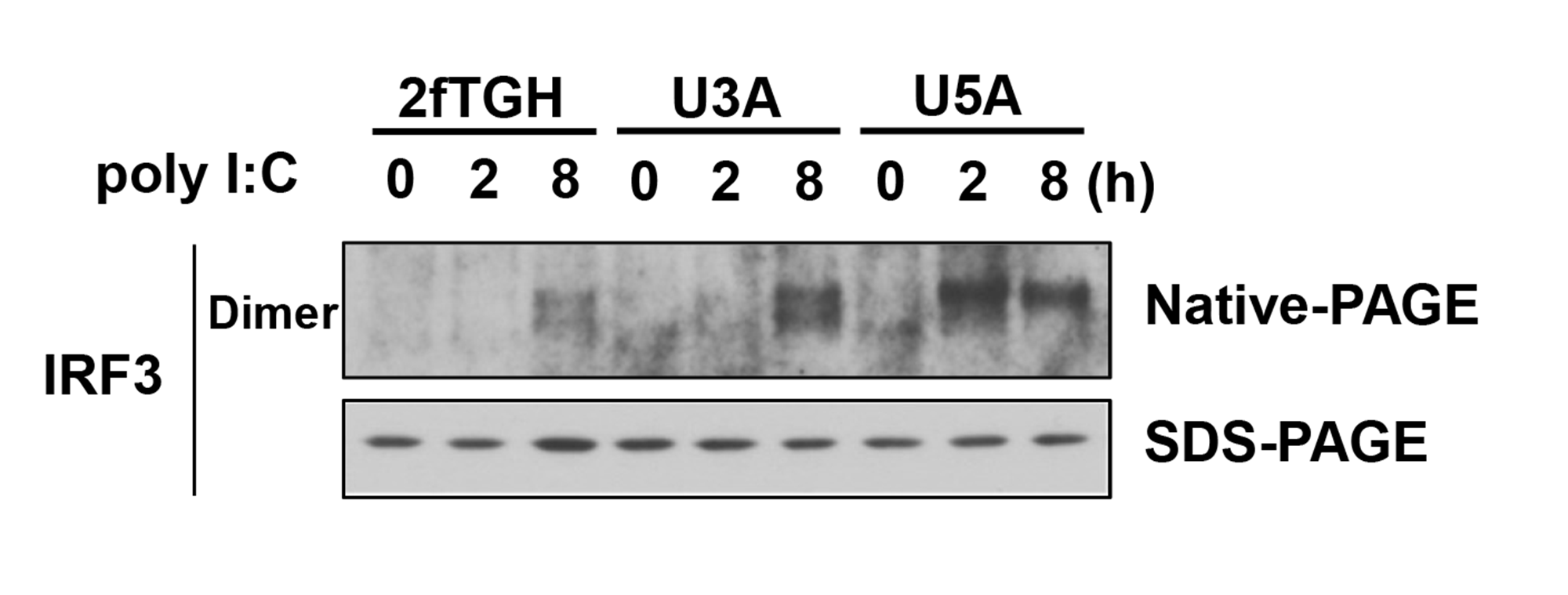

Supplement: S3 Fig — 2fTGH, U3A and U5A cells were transfected with poly I:C (100 ng) for up to 8 h and then harvested, and the lysates were subjected to native-PAGE or SDS-PAGE. Immunodetection was performed using an anti-IRF-3 antibody. (TIF) [file pone.0163520.s003.tif]

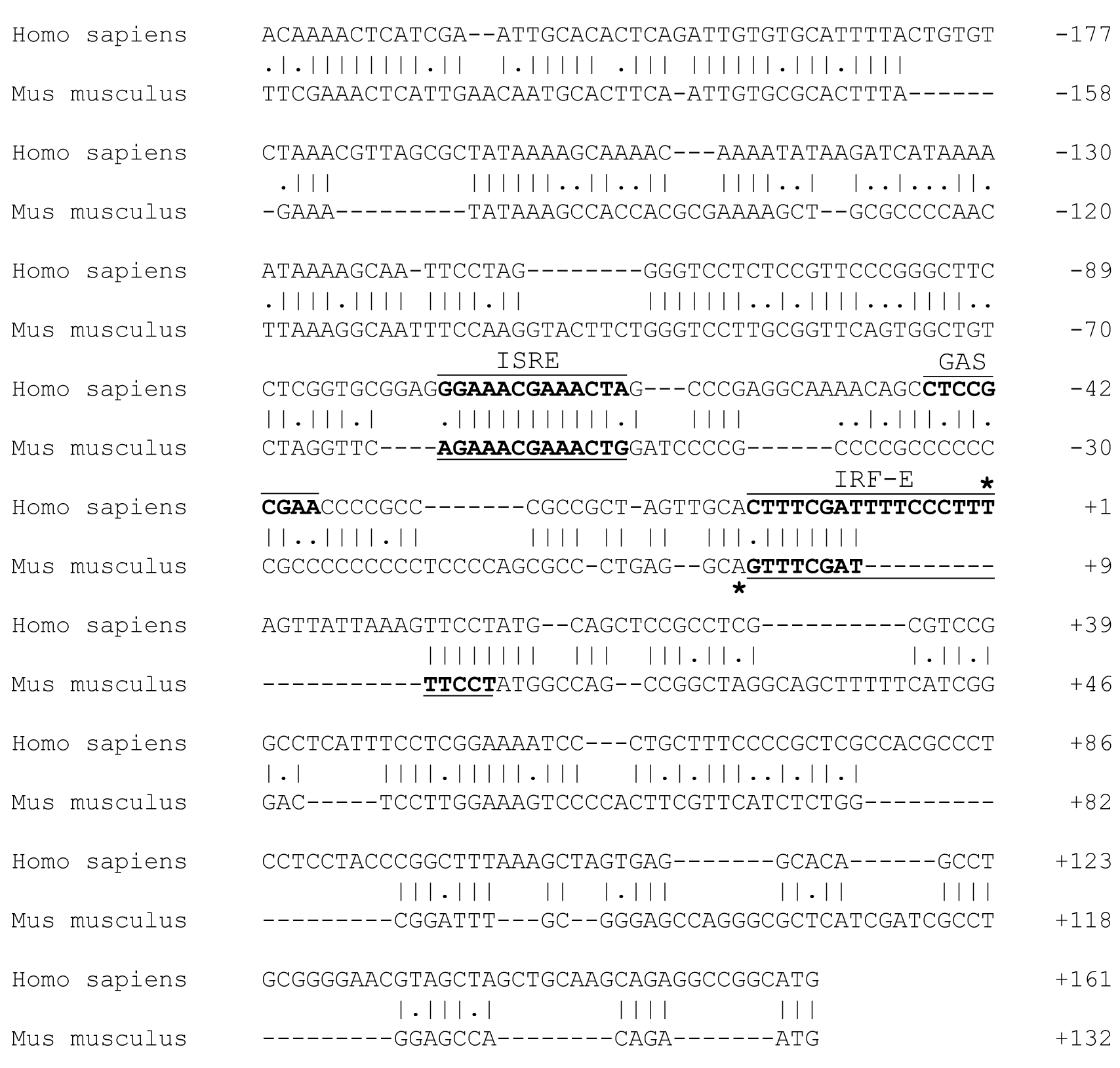

Supplement: S4 Fig — The proximal RIG-I promoters of human (-223 to +161) and mouse (-200 to +132) are shown. The putative consensus sequences of ISRE, GAS, IRF-E are highlighted. The asterisks indicate the transcription start site. (TIF) [file pone.0163520.s004.tif]
